# Decontamination potential of date palm fruit via non-thermal plasma technique

**DOI:** 10.1038/s41598-022-22335-5

**Published:** 2022-10-15

**Authors:** Khaled Lotfy, Salem Al‐Qahtani, Nadi Al-Harbi, Karima El-Absy, Faisal Bu Shulaybi, Saeed Alali, Tamer Mashtoly

**Affiliations:** 1 Department of Biology, Faculty of Science, Branch of Tayma, University of Tabuk, P.O. Box 741, Tabuk, 71491 Saudi Arabia; 2 King Marriott Higher Institute of Engineering & Technology, Alexandria, Egypt; 3 Eco-Physiology Unit, Plant Ecology and Ranges Department, Desert Research Center, Cairo, Egypt; 4 Palm and Dates Research Center, Ministry of Environment Waters, and Agriculture, Al Mubarraz, 36321 Al-Ahsa Saudi Arabia; 5 Department of Plant Protection, Faculty of Agriculture, Ain Shams University, Cairo, 11241 Egypt

**Keywords:** Biophysics, Microbiology, Physics

## Abstract

The potential of the surface dielectric barrier discharge technique (SDBD) was evaluated to decontaminate the date palm fruit. Preliminary investigations emphasized that *Aspergillus niger* fungus was predominant in most date samples as a post-harvest infestation. The influence of SDBD techniques on the viability of *A. niger* isolated from date varieties was investigated and documented. Physical and chemical characterizations of treated dates were assessed, and statistical correlation coefficients were calculated and elucidated. A 4 log_10_ reduction of *A. niger* radial growth was observed at 3 min exposure/15 days of incubation. Simultaneous reductions in pH, water activity, and moisture content of treated dates were observed when compared to untreated dates. Statistical analysis showed a positive correlation between physical and chemical variables with the viability of *A. niger* in treated samples. Therefore, we believe that SDBD treatment will be a promising technique for decontaminating date fruits from attacked fungi, which will positively impact sustainable food security and consumer health.

## Introduction

The date palm (*Phoenix dactylifera*) is one of the most adapted plants in the desert due to its capability to tolerate high temperatures, droughts, and salinity^1^. Meanwhile, date fruits are distinguished by containing primary essential nutrients and supplements such as proteins, sugars, fatty acids, minerals, and vitamins^2^. Thus, date fruits represent an essential integrated diet for humans and sometimes animals in the Arabia region^1^. The Kingdom of Saudi Arabia is the second largest producer of palm dates globally, with an estimated annual production of 1.5 million tons after Egypt, the first producer with an annual production of 1.8 million tons (FAO, 2021). Great efforts were exerted to maintain the date palm quality and safety. Unfortunately, date palm fruit, whether pre-or post-harvest, is attacked by many pests such as fungi, bacteria, and insects, causing an economic loss for this strategic crop yield. Most of the dates offered in the Saudi market are consumed without any kind of treatment to reduce microbial contamination, which could be spread out during transportation, handling, storage, and production lines^3^.

Furthermore, high levels of converted sugars and moisture content along with the humid weather while harvesting and ripening render date fruit suitable for fungi infections^4^. *Aspergillus flavus*, *Aspergillus niger*, *Penicillium chrysogenum*, and *Rhizopus stolonifer* were the most found infectious mycotoxins in marketed dates^3^. Aflatoxins are specific mycotoxins produced in infected foods by *A. flavus and A. parasiticus,* which are well known as genotoxic and carcinogenic agents and pose a severe threat to human health^5^. Furthermore, demand for dates is normally influenced by a lack of product quality and safety, particularly if the dates appear in exotic black due to external or internal fungus growth.

Chemical synthetic fungicides prepeared from organo-thiocarbamates and dithiocarbamates, such as Ziram®, Thiram®, Ferbam®, Maneb®, and Propineb®, were effective against pre- and post-harvest pathogenic fungi. Over the last decade, methyl bromide has been shown to be effective as an ozone-depleting fumigant in controlling post-harvest pests that attack commodities in storage^6^. The extensive use of methyl bromide resulting in serious impacts on the environment and human health^7^.

A lot of concerns about the use of methyl bromide in storage or pre-shipment have been raised worldwide, even in developing countries. Therefore, looking for alternative substances or techniques that may be used as decontaminants to protect commodities after harvest has become imperative^8^. A failure of the temperature-based technique was documented as an alternative disinfectant in combating pathogenic fungi due to its adverse effects on nutritional value as well as agricultural product quality^9^. Hence, recently, many non-thermal decontamination techniques such as ultraviolet, ultrasound, and non-thermal plasma have been developed to overcome the side effects of traditional thermal decontamination methods^10^. Recently, the interest in plasma technology has increased due to its special features such as quick treatment, low cost, high impact on microbial inhibition, no use of toxic gas, no harmful by-products, and safety to humans^11^.

Non-thermal plasma is considered a modern decontamination technique distinguished by the emission of charged reactive particles of oxygen, nitrogen, ozone, and ultraviolet radiation that exhibit effectiveness in microbial inhibition, preserving food commodities and agricultural products^12^.

Interestingly, the application of non-thermal plasma in agriculture has been increased in treating seeds, water, soil, and food manufacturing as well^13,14^. Non-thermal plasma stops fungi from growing by breaking conidia and cell membranes, which causes cytoplasm to leak out of fungal cells^15^.

Non-thermal plasma has more remarkable power to inhibit gram-negative bacteria than gram-positive bacteria due to the different composition of the cell envelope for them^16,17^. The break of conidiophores and vesicles, the crevasse of cell walls and cell membranes structures, and the cytoplasm leak occur for fungal cells after non-thermal plasma treatment^18^. Recently, it has been shown that non-thermal plasma has a new effect on how mycotoxins decompose^19^.

The physicochemical and organoleptic measures, such as color, pH, acidity, and electrical conductivity, of fruit and vegetable juices treated with non- plasma for microbial decontamination did not change^20–23^. Also, the non-thermal plasma treatment had no effect on the dry matter or pH of the fresh tomato juice^22^.

Surface dielectric barrier discharge (SDBD) has recently received broad interest in many applied sectors, especially in the decontamination of products. SDBD is composed of two metal electrodes separated by a dielectric material^24,25^. Plasma is generated at the electrode-air interface and usually emerges in the form of arrays of many unique sub-millimeter discharges^26,27^. For food processing, SDBD can be applied to ambient air so that there is no direct contact between the food and the active plasma area. Therefore, only neutral species participate in the decontamination process, and foods are not affected by UV or charged particles, ensuring optimal processing conditions^24,25^. The concern about the undesirable effects of the generated free radicals in the air using SDBD^28^ can be overcome by not contacting the products with the active plasma area. Due to its unique design, low operating cost, not using any toxic gas, and uniform distribution of neutral reactive species over the whole sample, SDBD is considered an economical and environmentally friendly alternative to traditional food processing technologies^24,25,29^.

Generating an optimum and economic ozone dose is the critical outcome of using SDBD in the food processor^29^. Artificially inoculated cherry fruits revealed that inhibition of fungal spores by the SDBD prolongs their shelf life. The enhancement of the fight against infections can be attributed to the stimulating defense responses in plant tissues that occur during the pretreatment of fruits or vegetables using SDBD^30^. The inhibition process using dielectric barrier discharge inhibited the growth of bacteria and mold species encountered in spoiled produce (green beans, grape tomatoes, lettuce, and strawberries), resulting in a more significant than 5 log_10_ decrease of microbial colonies^29^.

This work aimed to use the surface dielectric barrier discharges (SDBD) system in palm dates decontamination. Investigate the influence of the non-thermal plasma on the vitality of *A. niger* spores were isolated from some date varieties. Explore the bioactive site of H_2_O_2_ with amino acids of *A. Niger* using molecular docking. Assess the positive and negative impacts of SDBD treatment on the physicochemical properties of date varieties. However, to our knowledge, no previous studies have been found regarding the effect of SDBD on palm date varieties.

## Experimental setup

### Plasma system

**Figure 1:**
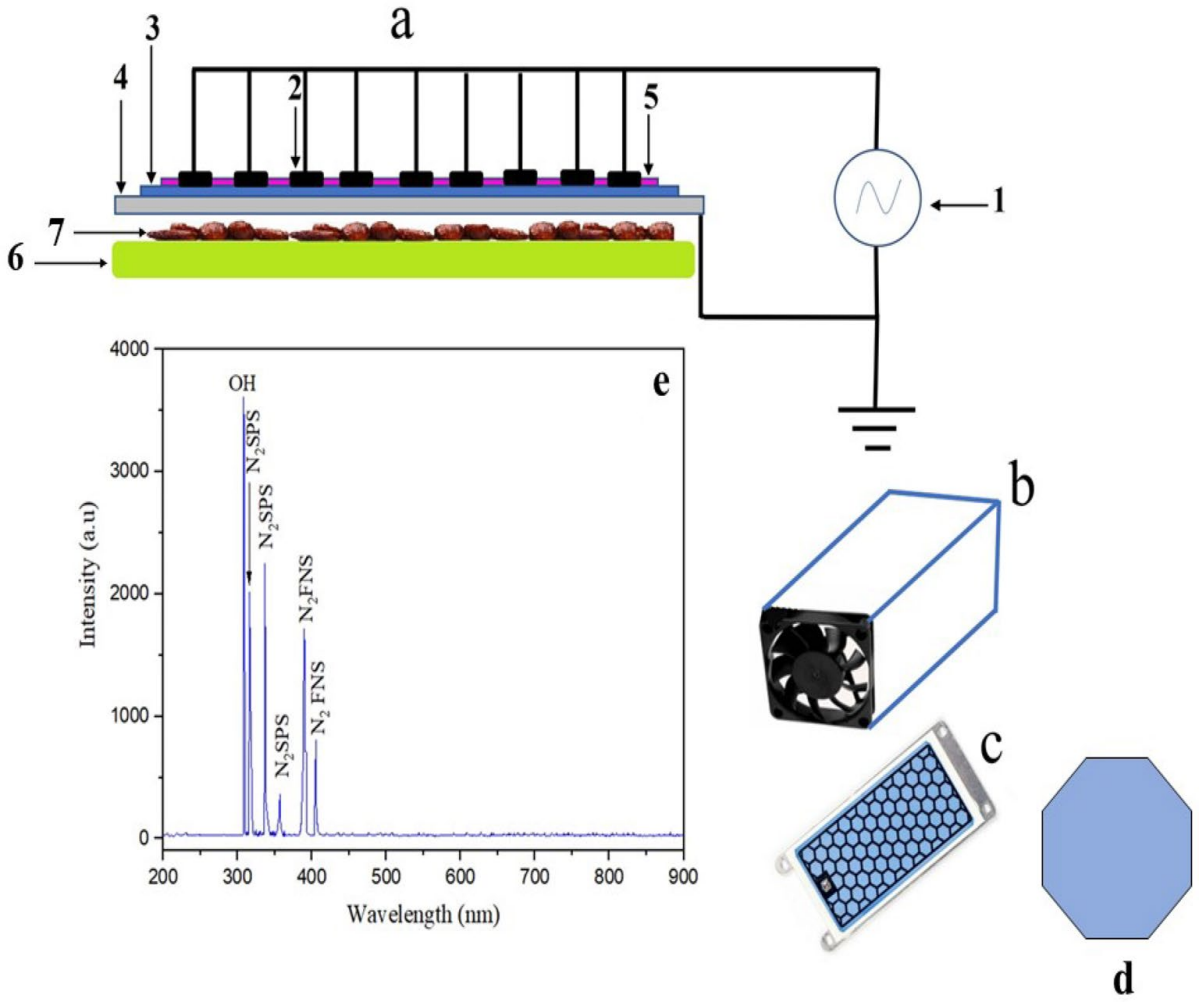
SDBD electric circuit (a); [Power supply (1), HV electrode(2), Dielectric barrier (3), Ground electrode (4), Plasma (5), Sample holder (6) Palm date samples (7)], Teflon box contains the SDBD system (b), honeycomb geometry of SDBD (c), the cell geometry of honeycomb (d), and the emission spectrum of the plasma from SDBD in the range of (200–900 nm) (e).

The surface dielectric barrier discharges (SDBD) electric circuit (Fig. 1a) has been adjusted in a Teflon box (24 × 12 × 8 cm) (Fig. 1b). To reduce the temperature of the produced plasma, the Teflon box has been attached to a cooler fan with a speed of 200–1200 rpm. The opposite side of the cooling fan was closed using a cork plug after the sample was placed inside the Teflon box. Commercially available SDBD with an alumina dielectric and printed electrodes in a honeycomb geometry (Fig. 1c) were used in this system^31^. The cell geometry of a honeycomb has been illustrated in Fig. 1d. The honeycomb breadboard is made of a 9 × 4 cm^2^ alumina dielectric surface, 1 mm in thickness, with an aluminum esagonal exposed electrode with a diameter of the circumscribed circumference of 7 mm. The ground electrode is a metallic plain plate. The plasma was generated by a high voltage (HV) power supply working in a power range between 8 and 30 W^32^ and a frequency of the order of 10 kHz. The applied HV is between 5 and 10 kVpp^33,34^. The SDBD is generated by the flowing air into the Teflon box via the cooler fan. The emission spectra of generating plasma over a broad wavelength range of 200–900 nm was detected using HR4000CG-UV-NIR (Ocean Optics) spectroscopy. Moreover, the spectrometer consists of a lens connected to a detector via a fiber optic cable (QP400-2-SR) with a diameter of 400 mm.

### Ethics approval and consent to participate

The collection of palm date fruit samples from the local markets in the Al-Ahsa region, Saudi Arabia was permitted by local shopkeepers on a survey. The plant collection and the study complied with local and national (Saudi Arabia) regulations. The study was approved by the institutional research ethics committee of the Palm and Dates Center, Ministry of Environment, Waters, and Agriculture, Al-Ahsa, Saudi Arabia, and written informed consent was obtained from each participant. All the methods in this manuscript were carried out in accordance with relevant guidelines and regulations.

### Sample collection for fungi inhibition using SDBD

Twenty-five samples of dates (2 kg/sample-*Tamr* stage) were randomly selected from seven date varieties; *Ajwa*, *Rashodia*, *Nabtet Ali*, *Magdol*, *Shalabi*, *Sukkari*, and* Khudari* from the local markets in the Al-Ahsa region, Saudi Arabia. Special paper bags were used for sampling^35^; they were sterilized to correct for external contamination. The samples were transferred promptly within 15 min in an equipped ice box container aseptically to the Dates Quality and Safety Unit at the Palm and Dates Center for microbiological inspection.

### Isolation and identification of fungi associated with dates

To separate the obviously infected dates, 100 g of each sample were subjected to physical examination for fungi^36^. Moreover, 10 individual dates from each sample were surface sterilized by 2% sodium hypochlorite solution for 2 min, washed twice with sterile double distilled water, and then cut into small pieces (1 cm^2^/piece) under aseptic conditions. Each small piece was cultured separately on Potato Dextrose Agar (PDA) plates and incubated for 5 days at 26 to 28 °C ± 1; RH 72–75 ± 1. Single hyphen tips of each different shape or color were subjected to sub-culturing under the same previous conditions and repeated until they had pure fungal growth. The purified isolates were manually identified^37,38^ and then maintained at 4 °C until used. The fungal species which were isolated from all the varieties of palm dates are *Aspergillus niger*, *Fusarium*, and *Penicillium*.

### Decontamination treatment using SDBD system

In this work, *A. niger* represented the predominant isolated among fungi. A glass slide was sterilized with ethanol 70%, and wiped with a sterilized towel to be ready for the decontamination of date samples. In all runs, a 5 mm disc of *A. niger* was transferred onto the pre-sterilized glass slide*.* The individual disc of spore fungi was put on the sample holder 0.5 cm apart from the electrode for five different exposure time: 1, 1.5, 2, 2.5 and 3 min have been operated separately to evaluate the impact of the SDBD system in inhibiting *A. niger* spores and determine the proper decontamination time*.*

### Sample collection for palm dates treatment using SDBD

Samples of the seven commonly used date palm varieties were subjected to a decontamination process in food science manufacturing at the Date Palm Research Center Al-Ahsa, Saudi Arabia. Samples were inspected and deteriorated fruits were eliminated and disposed of. Healthy fruits were kept at 5 °C for about 24 h. Date samples were treated using the SDBD system for 3 min dependent on the electrode coverage area and sample size, after the preliminary examination suggested this was the proper decontamination time. Ten to fifteen samples of each date cultivar were treated by exposing them to SDBD, 0.5 cm away from the electrode.

### Molecular docking

H_2_O_2_ was obtained as a sdf file from PubChem and converted to a PDB file using PyMOL 2.5 software. The *A. niger* proteins (PDB: 3K4Q, 1KS5) were obtained from the Protein Data Bank (PDB)^39^. Before docking analysis, interacting ligands and water molecules were removed from the used proteins. Autodock tools were used to perform the docking analysis. In the present study, LigPlot+ was used to identify interacting residues and the interacting bonds between the target and hydrogen peroxide^40^.

### Physicochemical measurements

The moisture content of control and treated samples of dates with SDBD were determined using AOAC procedures^41^. Water activity of date samples were measured using a water activity meter (AQUA Lab aw, Decagon). Total soluble solids (TSS) of date samples were determined as % using a hand refractometer (Digital Refract meter, SPER Scientific), SPER SCIENTIFIC LTD, Laboratory Digital Refractometer (Brix), Type: 300034 Brix nD. A pH meter was used to estimate the pH values of treated and untreated dates. Texture profile analysis of palm date samples was measured using a texture analyzer (TA. XT plus, Stable Micro Systems Ltd., Surrey, UK), 75-mm-diameter disk plunger (P 75)^42^.The force was measured by compacting the date fruits while they were placed horizontally^43^. The squeezing procedure included two bites to get the texture profile analysis properties, which included basic properties such as hardness, chewiness, resilience, cohesiveness, gumminess, adhesiveness, and springiness. Color reflectance values of date samples (L*, a*, b*, c* and h*) were measured using a Hunter Minolta Chroma meter (Hunter Associates Laboratory Inc. Restan VA., USA)^44^.

### Statistical analysis

Data values are expressed as Mean ± standard error (SE). Analysis of variance (ANOVA) was performed to determine the effect of treatment using SPSS software package (version 20). The Least Significance Difference (LSD) was conducted when a significant difference at *P* ≤ 0.05 was statistically occurred. The principal component analysis was applied for a better understanding of the relationship among studied measures across palm date varieties that were both untreated and treated with SDBD, using Origin Pro 2021 version b 9.5.0.193 computer software program.

## Results

### Plasma diagnostics

The emission spectrum of the SDBD in the range of (200–900 nm) was measured using optical emission spectroscopy (OES), as can be seen in Fig. 1e. The molecular spectra of a nitrogen second positive system (N_2_ SPS) were shown at 316, 337, and 357 nm. However, the peak of nitrogen first negative system (N_2_ FNS) was shown at 389 and 405 nm. Furthermore, the OH radical has emerged at 308.9 nm. It is well known that hydroxyl radical (OH) negatively affects the microorganisms' cell membranes^45^. On the other hand, in this work, the smell of ozone was easily distinguished while the SBDB device was running. The produced reactive radicals by non-thermal plasma generate an oxidative stress response driving harmful oxidative cell spoilage^46^, followed by inhibition of microorganisms viability and vitality^47^. By conserving specific bioactive chemicals, non-thermal plasma may be a useful technique for extending the shelf life of vegetable juice^23^.

However, *A. niger* and palm date samples have been treated without operating the SDBD system to study the effect of the cooler fan air (results not included). The results revealed no impact of the cooler fan air on the treated samples.

### Inhibition of *A. Niger* using SDBD system

**Figure 2:**
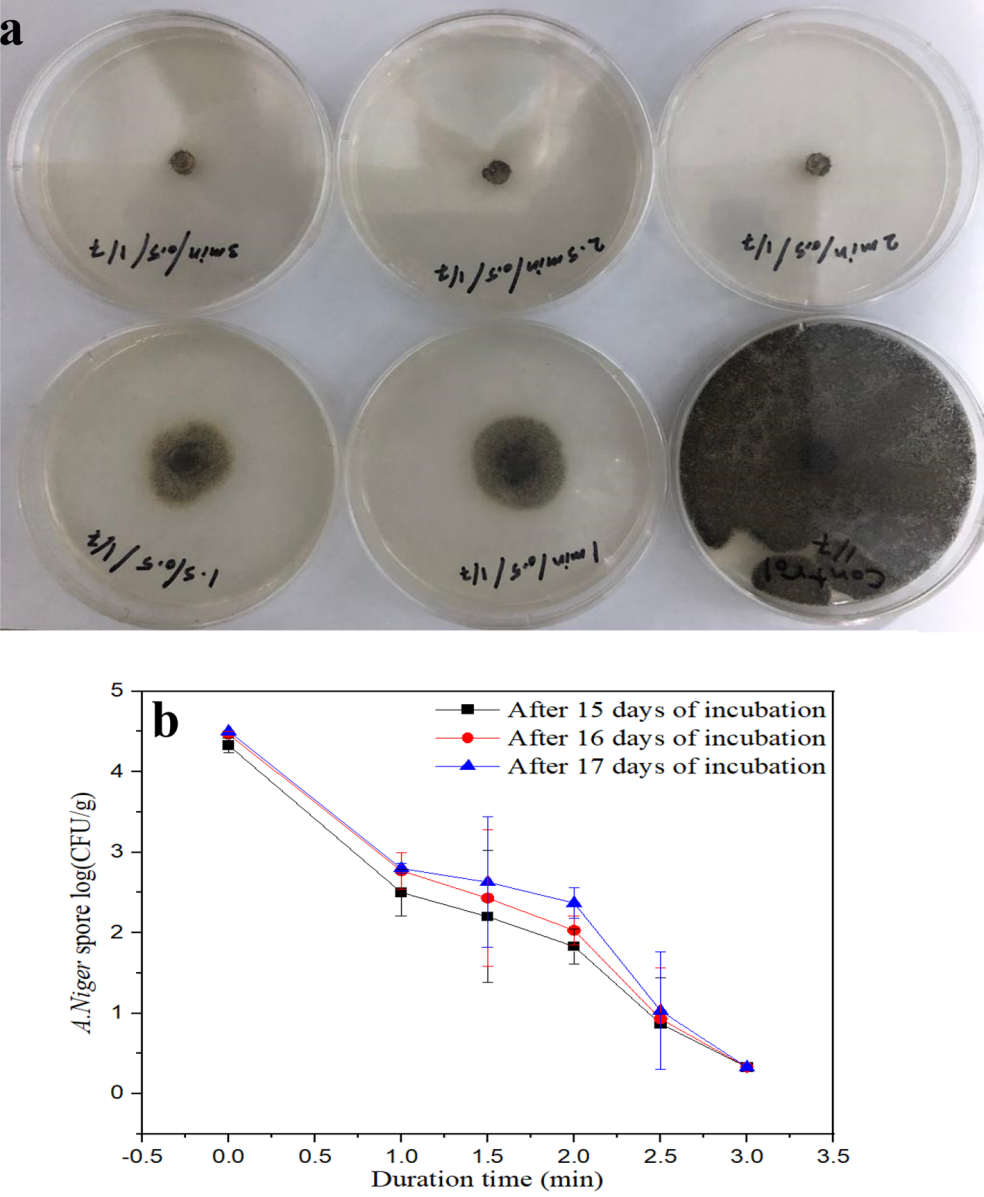
Inactivation of *A. niger* using SDBD at different treatment time; (**a**) *A. niger* spore after 2 days of incubation and (**b**) *A. niger* spore log_10_ (CFU/g) after 15, 16 and 17 days of incubation, P value = 0.00.

The effect of SDBD on *A. niger* after 48 h of incubation at different treatment times is illustrated in Fig. 2a. The increase in the treatment time led to the efficacy of SDBD to inhibit *A. niger*. Moreover, after 15 days of incubation, the survivor numbers (CFU/g) of *A. niger* in treated samples were decreased with an increasing exposure time to plasma (Fig. 2b). Simultaneously, for untreated samples, the survivor numbers of *A. niger* reached 4.33 ± 0.09 CFU/g, while for plasma-treated *A. niger*, with an increase in the exposure time from 1 to 3 min, the cell numbers decreased from 2.50 ± 0.29 to 0.33 ± 0.03 CFU/g, respectively. The reduction of *A. niger* was 1.83 log_10_ after 1 min of exposure, and it increased to 4 log_10_ after 3 min of exposure. The results revealed significant differences (p < 0.05) in the survivor numbers of *A. niger* between the exposure time of plasma.

After 16 and 17 days of incubation, we find that the survivor numbers of *A. niger* for untreated samples are 4.47 ± 0.03 and 4.50 ± 0.00 CFU/g, respectively. Moreover, as the exposure time is increased from 1 to 2.5 min, the survivor numbers of *A. niger* increase with the incubation period. However, at an exposure time of 3 min, the survivor numbers of *A. niger* did not increase with the incubation period. The results showed that the growth rate of *A. niger* cells after 16 and 17 days of incubation gradually decreases with increasing exposure time from 1 to 3 min, and it became zero at 3 min. Statistically, the results displayed no significant difference (p > 0.05) on the growth rate of *A. niger* after 16 and 17 days of incubation. These results indicated that the optimum conditions for inhibition of *A. niger* using SDBD are at an exposure time of 3 min. Similar results were reported, it was found that, a considerable drop in the overall viable count was seen after 30 s of plasma treatment and in the yeast and mold count after 60 s^23^.

Recently, it was proved that non-thermal plasma virtually inhibited *Aspergillus parasiticus* in conidia and hyphae within a few minutes of treatment^48^. Complete inhibition of the extracted *A. niger* from palm dates was recorded after 9 min at a 3.5 L/min argon flow rate using a DBD system^49^. However, complete inactivation of the formed *Aspergillus* sp. on saffron after 15 min plasma exposure at 60 W using radio-frequency plasma^50^. After 20 min of plasma exposure, *P. buchwaldii, P. expansum*, and *P. bialowiezense* were reduced by 2.6, 2.2, and 1.9 log10, respectively^51^. It has been found that after 60 s of DBD treatment, the cell walls and cell membrane structures of *Ascochyta pinodella* and *Fusarium culmorum* were destroyed, resulting in leakage of cytoplasm, while the cells were flattened after 180 s^52^. From previous and current results, it can conclude that, the effect of plasma on the fungi depends on many factors such as, the supplied gas type, the device design, the distance between the electrodes and the sample, the plasma power, the plasma temperature, and the exposure time.

The antimicrobial efficacy of plasma can be explicated as the destruction of the cellular structure and cell leakage^53^ or the destruction and cracking of the cell walls or cell membrane of microorganisms^54^, which occurs by free radicals, UV-emitting species, and charged particles after nonthermal plasma treatment. On the other hand, hydroxyl radicals can destroy microbial cells due to their high electronegativity, making them potent oxidizing agents. The various enzymes, proteins, and unsaturated fatty acids of the membrane lipid bilayer are considered the main targets of oxidative processes for germ^55^. Also, hydrogen peroxide has high microbicidal effects^56^. Hydrogen peroxide could be produced in a non-thermal plasma environment due to the reaction between two hydroxyl radicals^57^ and it would act as a decontaminant^56^. However, the generated ozone by the DBD over 3 and 15 min resulted in at least a 1 and 4 log_10_ reduction in microbial colonies present in the spoilage inocula, respectively^29^. Furthermore, atomic oxygen, O(1D), has been shown to play an important role in the inhibition of *Aspergillus oryzae* and *Penicillium digitatum*^58^.

### Molecular docking

**Figure 3:**
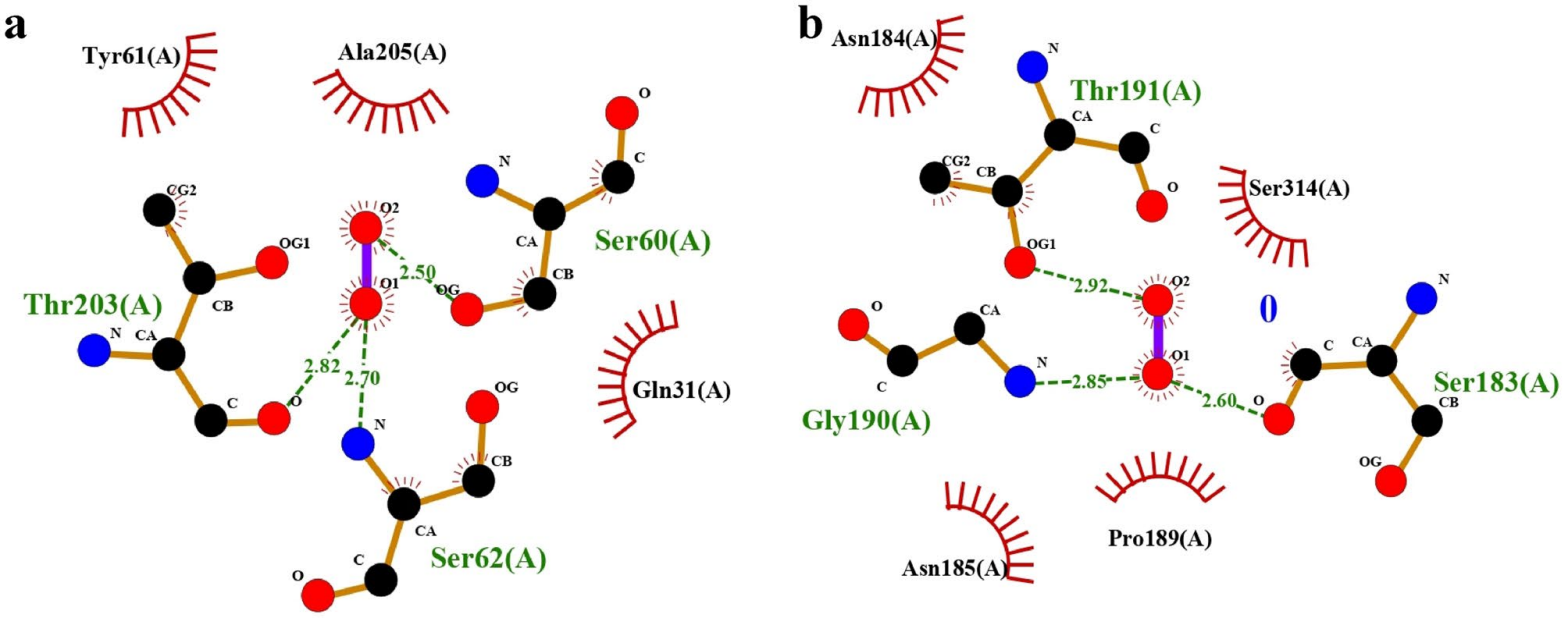
The interaction between H_2_O_2_ and the amino acid residues of *A. niger* (**a**) 1KS5 and (**b**) 3K4Q.

Molecular docking was used to investigate the mechanism of interaction between the active plasma species (H_2_O_2_) and the two enzymes of *A. niger* (PDB: 3K4Q, 1KS5). The docking simulation of H_2_O_2_ against *A. niger* enzyme (PDB: 1KS5) has a binding affinity of -3.29 kcal/mol. Figure 3a illustrated that H_2_O_2_ has hydrophobic interaction with Gln31, Ala205 and Tyr61. On the other hand, three hydrogen bonds with a length of 2.82, 2.70 and 2.50 Å were generated between H_2_O_2_ and Thr203, Ser62 and Ser60 respectively. The docking simulation of H_2_O_2_ against the *A. niger* enzyme (PDB: 3K4Q) has a binding affinity of -3.57 kcal/mol. Moreover, a hydrophobic interaction between H_2_O_2_ and Asn185, Pro189, Ser314, and Asn184 can be observed in Fig. 3b. Also, H_2_O_2_ formed three hydrogen bonds with Thr191, Gly190, and Ser183, with a length of 2.91, 2.85, and 2.60 Å. Numerous hydrogen bonds were produced between H_2_O_2_ and *A. niger* enzymes, indicating powerful binding in these systems. The results showed the possibility of an interaction between the amino acid residues of *A.niger* enzymes by forming hydrogen bonds. Therefore, the produced hydrogen peroxide from non-thermal plasma can inhibit fungi, especially *A. niger* under study^59^.

### The effect of SDBD treatment on physicochemical properties of palm dates

#### Water activity and moisture of palm dates

**Figure 4:**
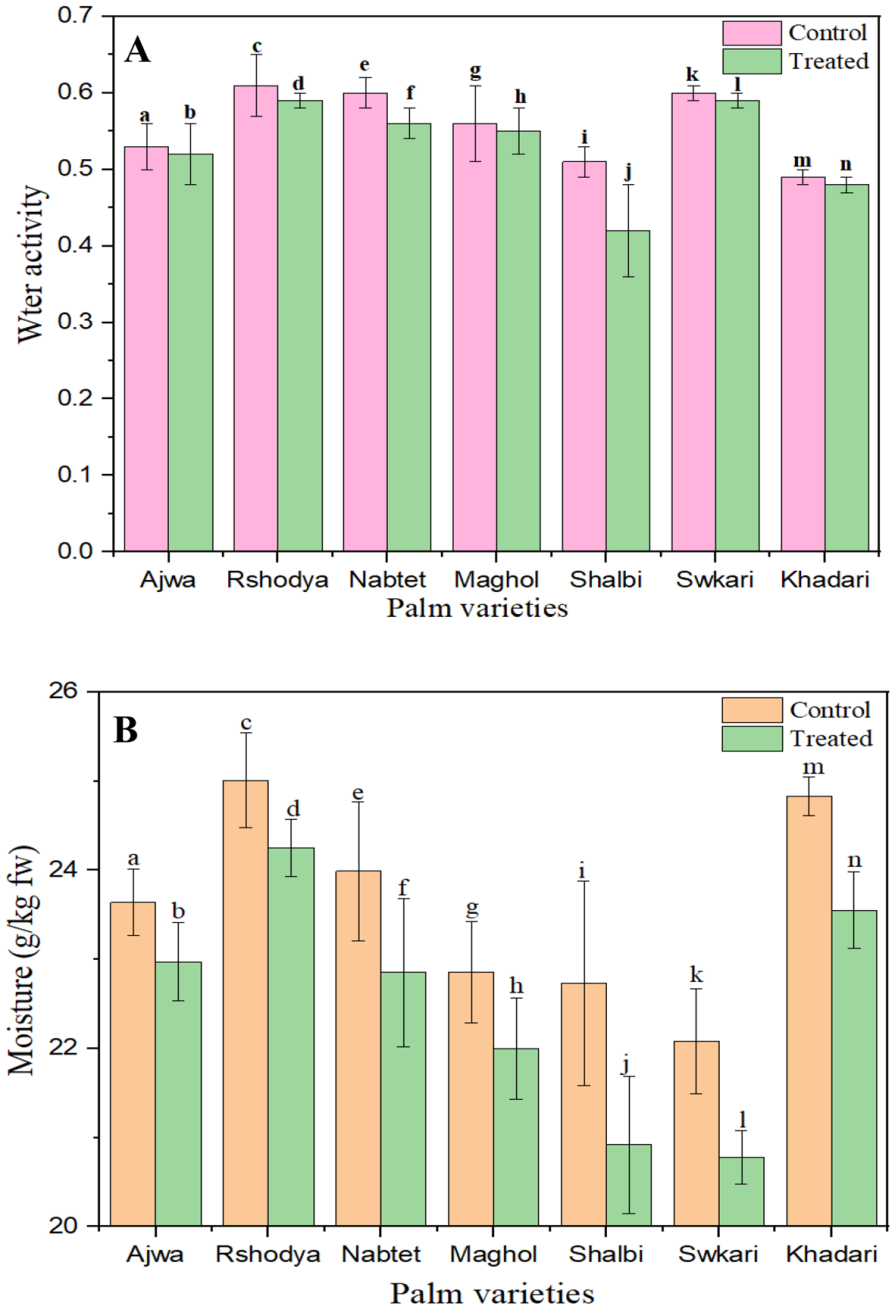
The influence of SDBD on Water activity, P value = 0.016 (**A**), and Moisture, P value = 0.002 (**B**) of Palm dates varieties.

It is known that the decontamination methods play a significant role in the value of water activity and moisture^60^. Changes in the water activity and moisture of seven types of palm dates treated with SDBD for 3 min are shown in Fig. 4A and B. From these results, there was a significant difference (p < 0.05) among palm date varieties in the water activity and moisture values. After plasma treatment, the water activity and moisture values of palm date varieties have decreased. The *Shalabi* date cultivar had the highest decreases in the water activity and moisture values, while the *Ajwa* date cultivar had the lowest after plasma treatment. It found that, a 5% moisture loss within 4 s of treatment in cucumbers, carrots, and pear plants was observed^61^*.*

#### Total soluble solid (TSS)

**Figure 5:**
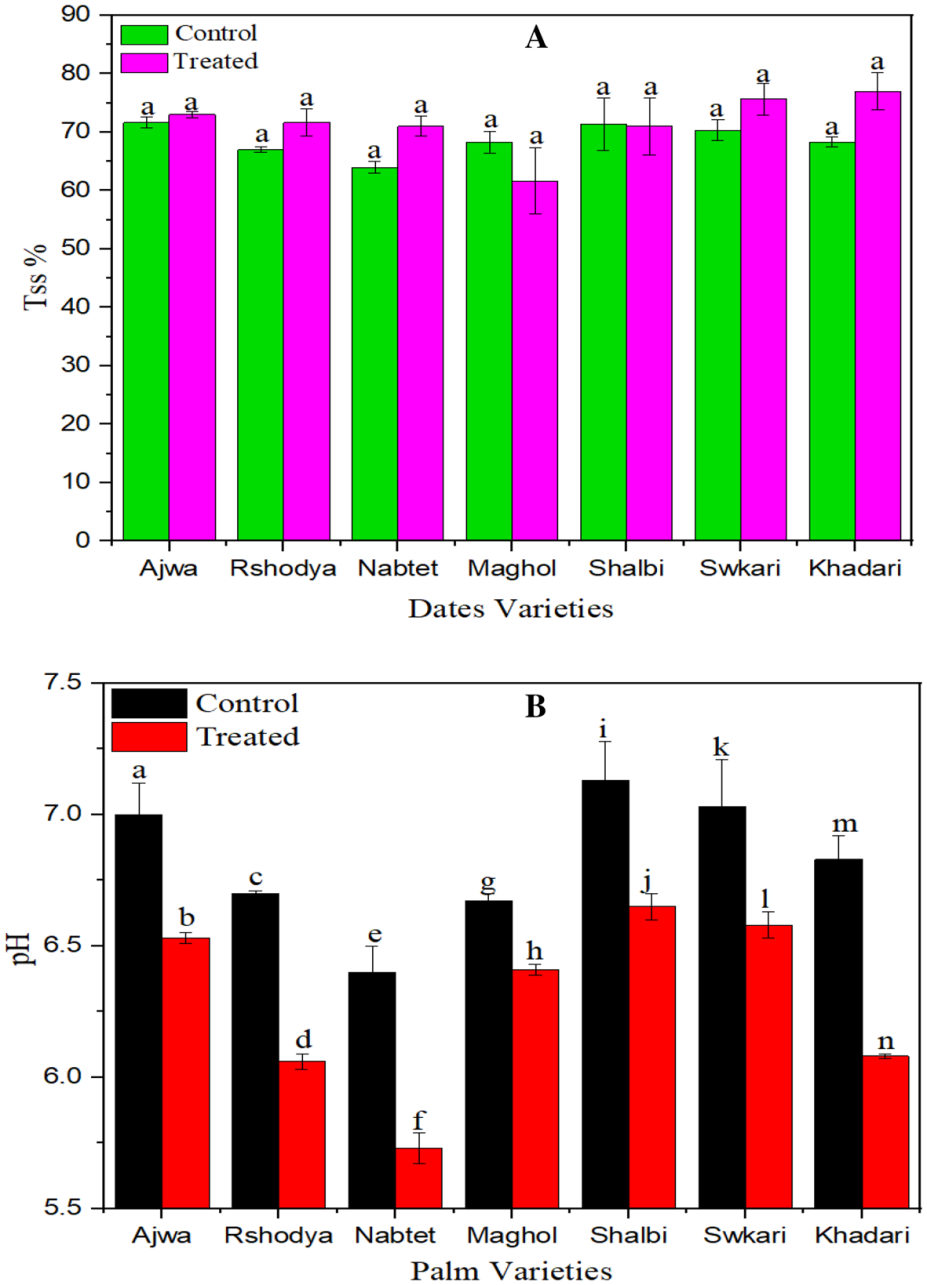
The influence of SDBD on TSS, P value = 0.70 (**A**), and pH, P value = 0.00 (**B**) of Palm dates varieties.

Total soluble solid (TSS) of the current palm dates has not been affected by SDBD treatment. Data illustrated in Fig. 5A demonstrated that there was no significant difference (p > 0.05) in TSS between treated and control palm dates. Similarly, it has been found that, non-thermal plasma treatment for 2.5 min had no significant effect on TSS of strawberry^62^. Further, atmospheric non-thermal plasma treatment for 10 min and 15 min had no significant effect on TSS of strawberry, but 30 min had a significant effect on TSS of strawberry^63^. In another study, no significant changes occurred in the solid soluble content of apple fruits after plasma treatment^64^.

#### pH

The effect of SDBD treatment on the pH values of palm dates is given in Fig. 5B. For all palm date tested varieties, pH value has decreased after SDBD treatment for 3 min. A significant difference (p < 0.05) in pH between treated and untreated palm dates was found. *Khadri* date fruit had the highest decrease in pH (from 6.83 ± 0.09 to 6.08 ± 0.01), while the *Maghol* date fruit had the lowest decrease (from 6.67 ± 0.03 to 6.41 ± 0.06). However, decreasing limits in pH among all treated palm date varieties were comparatively small.

Moreover, it has been proved that a decrease was found in the pH of strawberry after non-thermal plasma treatment^63^. Also, an increase was observed in acidity in terms of a pH decrease in treated brown rice with plasma^65^ and similarly occurred in treated orange juice with non-thermal plasma^66^. The non-thermal plasma might have a more significant effect on the pH value of liquid specimens than solid ones^67^ due to the interaction between the moisture phase of liquid samples and generated reactive gases. In contrast, acidic compounds are generated just on the surface in the case of solid specimens. The pH of the control samples and tomato juice exposed to non-thermal plasma for 30 and 60 s did not differ significantly^23^. For plasma treatments lasting 120 and 300 s, the pH increase was somewhat greater in tomato juice^23^.

#### Texture

Texture is a crucial quality character utilized in processing food to evaluate the quality of products. The hardness of fruits and vegetables is considered the essential character texture to determine the freshness of foods^68^. Texture profile analysis was conducted in dates fles^69^.

**Figure 6:**
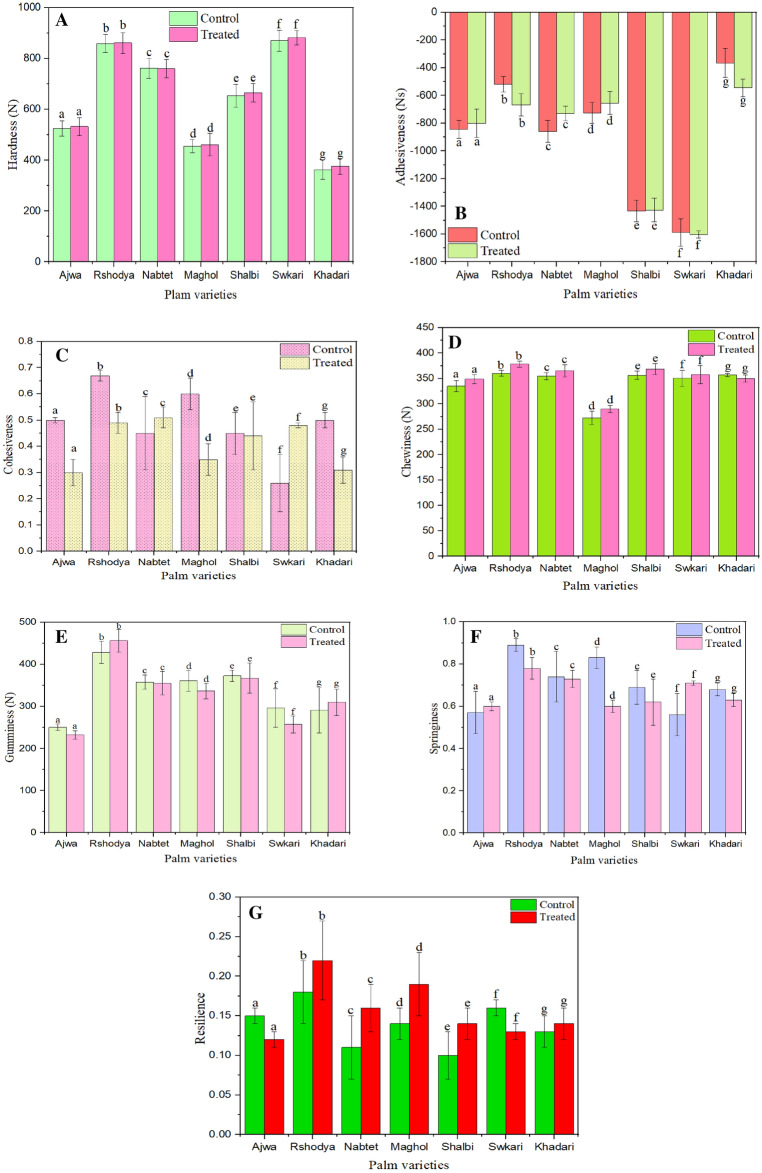
The influence of SDBD on texture of palm dates (**A**) Hardness P value = 0.06, (**B**) Adhesiveness P value = 0.75, (**C**) Cohesiveness P value = 0.06, (**D**) chewiness P value = 0.07, (**E**) Gumminess P value = 0.69, (**F**) Springiness P value = 0.24, (**G**) Resilience P value = 0.183.

The hardness of control palm dates ranged from 362.50 ± 9.67 N up to 870.65 ± 6.24 N. However, the hardness of treated palm dates using SDBD plasma for 3 min ranged from 375.55 ± 5.01 N up to 881.76 ± 8.20 N, as shown in Fig. 6A. The hardness of palm dates has decreased after SDBD treatment for *Nabtet Ali *date cultivar from 761.70 ± 3.03 to 759.51 ± 6.26. However, the hardness values were increased for the rest of the palm date treated varieties. Statistical analysis showed that there was no significant difference (p > 0.05) in the hardness between treated and untreated palm date varieties.

The adhesiveness values of *Swkari* and *Khadari* varieties decreased after SDBD treatment from − 1587.88 ± 99.51 to − 1602.65 ± 27.15 Ns and from − 364.82 ± 99.41 to − 544.35 ± 64.70 Ns respectively. On the other hand, the adhesiveness values of the rest of the date varieties were increased as can be seen in Fig. 6B. Nonetheless, there is no significant difference (p > 0.05) in the adhesiveness between treated and control palm dates.

Figure 6C elucidated that cohesiveness values of untreated samples ranged from 0.26 ± 0.11 to 0.67 ± 0.02, while they were ranged from 0.30 ± 0.05 to 0.51 ± 0.04 for treated samples. The cohesiveness of palm date varieties has increased after SDBD treatment, for* Nabtet Ali* date cultivar increased from 0.45 ± 0.14 to 0.51 ± 0.04 and for *Sukkari* from 0.26 ± 0.11 to 0.48 ± 0.01. However, it has decreased for the rest of the palm date treated cultivars.

Regarding chewiness values, which appeared in Fig. 6D, an increasing trend was noticed after SDBD treatment for all palm date varieties except *Khadari*, which decreased from 356.63 ± 3.48 to 350.13 ± 7.87 N. Chewiness values ranged from 272.19 ± 13.38 to 360.12 ± 07.03 N for control samples, while they ranged from 290.08 ± 07.35 to 378.34 ± 06.09 N for treated ones. Despite that, there is no significant difference (p > 0.05) in the chewiness between treated palm dates using SDBD and untreated ones.

The gumminess of palm dates increased after SDBD treatment for *Rashodia *from 428.79 ± 26.65 to 456.52 ± 27.31 and for *Khadari* from 291.26 ± 53.81 to 310.29 ± 31.52 but decreased for the other palm date types as apparent in Fig. 6E. The gumminess values of untreated samples ranged from 250.77 ± 8.02 to 428.79 ± 26.65, while they ranged from 232 ± 9.82 to 456.52 ± 27.31 for treated ones. Nevertheless, there is no significant difference (p > 0.05) in the gumminess values between treated and control palm date samples.

Moreover, the results of springiness indicate that, there was no significant difference (p > 0.05) in the springiness between treated palm dates and untreated ones. The springiness values decreased for all palm dates varieties except for *Ajwa* and *Swkari* varieties, it increased from 0.57 ± 0.00 to 0.60 ± 0.02 and from 0.56 ± 0.10 to 0.71 ± 0.01, respectively, as explained in Fig. 6F.

In the case of resilience, untreated samples ranged from 0.10 ± 0.02 to 0.18 ± 0.04, on the other hand, they ranged from 0.12 ± 0.00 to 0.22 ± 0.01 for treated ones. The resilience of palm dates decreased following SDBD treatment in *Ajwa* whereas, ranged from 0.15 ± 0.01 to 0.12 ± 0.00 and in *Swkari* ranged from 0.16 ± 0.02 to 0.13 ± 0.01 but increased for the other palm date types as apparent in Fig. 6G. Regardless, no significant difference exists (p > 0.05) between resilience values of treated and untreated palm date samples.

Statistical analysis demonstrated that no significant difference occurred in the texture of palm dates between treated and untreated samples. Simultaneously, most previous studies reported thatno significant differences appeared in texture change after non-thermal plasma treatment between control and treated samples of banana^70^, of radicchio^71^, and of lettuce^72^.

#### Color

**Figure 7:**
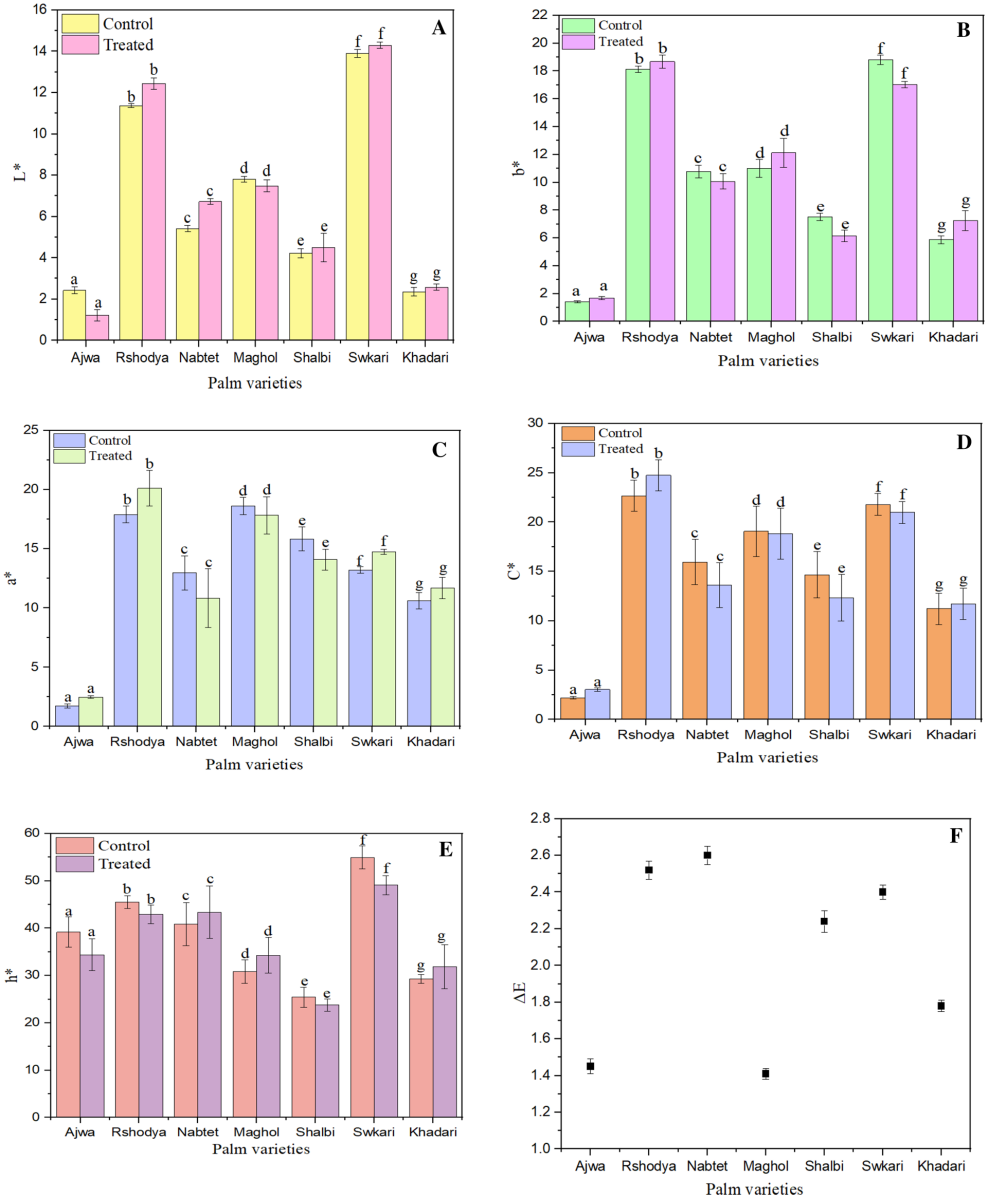
The impact of SDBD on color parameter of palm dates; (**A**) L*, P value = 0.08, (**B**) b*, P value = 0.76, (**C**) a*, P value = 0.82 (**D**) c*, P value = 0.74 (**E**) h*, P value = 0.57 and (**F**) ΔE.

It is noteworthy that L*, Chroma, and Hue food might be affected by the storage period, type of treatment, or both. The effect of SDBD treatment for 3 min on the color of current palm dates was illustrated in Fig. 7. The measured L* values of palm dates have decreased after SDBD treatment for *Ajwa* from 2.43 ± 0.17 to 1.22 ± 0.27 and *Maghol* from 7.81 ± 0.14 to 7.49 ± 0.29, but they have increased for the rest of the treated palm date types (Fig. 7A). While L* values varied from 2.36 ± 0.21 to 13.90 ± 0.18 for untreated samples and they varied from 2.58 ± 0.15 to 14.30 ± 0.15 for treated ones. No significant difference was shown (p > 0.05) in L* values between treated and control samples.

Moreover, b* values have increased after SDBD treatment for *Ajwa* from 1.41 ± 0.08 to1.69 ± 0.11, *Maghol* from 11.01 ± 0.65 to 12.12 ± 1.04, *Rashodia* from 18.13 ± 0.48 to 18.69 ± 1.04 and *Khadari* from 5.87 ± 0.29 to 7.25 ± 0.71 while, it has decreased for the rest of the treated palm date varieties, as illustrated in Fig. 7B. There was no significant difference (p > 0.05) in b* values between treated and untreated samples.

The a* values were in increasingly trend for all treated palm dates with SDBD treatment except for *Nabtet Ali*, *Maghol* and *Shalbi*, whereas they were in decreasing trend from 12.97 ± 1.44 to 10.83 ± 2.47, from 18.63 ± 0.73 to 17.83 ± 1.56 and from 15.84 ± 1.00 to 14.84 ± 0.89, respectively, as shown in Fig. 7C. Even so, there was no significant difference (p > 0.05) in a* values between treated palm dates using SDBD and control ones.

In addition, from Fig. 7D, illustrated the measured C* values of treated palm dates varieties using SDBD for 3 min, the results indicate that, C* values have increased after SDBD treatment for *Ajwa* from 2.21 ± 0.15 to 3.05 ± 0.17, *Rshodya* from 22.67 ± 1.59 to 24.75 ± 1.59 and *Khadari* from 11.22 ± 1.57 to 11.71 ± 1.57, while it was decreased for other treated palm date varieties. Nevertheless, no significant difference was shown (p > 0.05) between treated and untreated palm dates.

From Fig. 7E, hue values (h*) increased after SDBD treatment for *Nabtet Ali* from 40.85 ± 4.51 to 43.37 ± 5.55, *Maghol* from 30.84 ± 2.51 to 34.27 ± 3.76 and *Khadari* from 29.27 ± 0.94 to 31.89 ± 4.67 but decreased for the rest of the treated palm date varieties. The results revealed no significant difference (p > 0.05) in hue values between treated and untreated palm dates.

It was reported that the total color difference(ΔE) could be classified as follows; highly observable (ΔE > 3), observable (1.5 < ΔE < 3), and unobservable (ΔE < 1.5)^73^. Thus, we could realize that the total color difference might be unobservable for *Ajwa*, *Maghol*, and *Khadari* varieties. The results indicated that, no significant difference in the total color difference between treated and untreated palm date varieties. Similarly, no significant difference was determined in color change after non-thermal plasma treatment between control and treated samples of banana^70^ and treated samples of chili pepper^74^. In contrast, a significant change of color has been observed after non-thermal plasma treatment between treated and untreated samples in lettuce^75^, in cucumber and carrot slices^61^, and in blueberries as well^76^.

### Principal component analysis

The association between measured variables was assessed using principal component analysis (PCA) across palm date varieties that were both untreated and treated with SDBD. The six PCAs for studied variables affected by untreated and treated palm dates using SDBD are given in Table 1, which had eigenvalues higher than one (eigenvalue > 1). Out of all the PCAs, the two main PCAs (PCA1 and PCA2) were kept for the final analysis because they both have the highest eigenvalues greater than one and explain more than 65% of the total variance of all analyzed variables in untreated and treated palm dates using SDBD, respectively. These results indicate the variability in treated palm dates using SDBD was higher than in untreated palm dates using SDBD and confirm that the testing variables were different in both treatments. In treated and untreated samples, the PCA1 explained 38.16% and 45.13%, while the PCA2 accounted for 27.65% and 20.51% of the total variation of analyzed variables, respectively. Thus, PCA1 and PCA2 can be used as the basis for assessing the relationship between investigated variables of untreated and treated palm date varieties. The PC1 and PC2 explained 31.00% and 21.00%^77^, 60.27% and 17.11%^78^ and 77.9% and 11.4%^79^ of the total variance of date palm fruit variables, respectively.

**Table 1:** Results of PCA in the first six PCAs for the studied variables during untreated and treated palm date varieties.

Variables	Untreated						Treated					
	PCA1	PCA2	PCA3	PCA4	PCA5	PCA6	PCA1	PCA2	PCA3	PCA4	PCA5	PCA6
Eigenvalues	5.72	4.15	1.99	1.56	1.15	0.43	6.77	3.08	2.66	1.47	0.64	0.38
Variance %	38.16	27.65	13.26	10.38	7.67	2.88	45.13	20.51	17.77	9.81	4.27	2.52
Cumulative%	38.16	65.81	79.07	89.45	97.12	100.00	45.13	65.63	83.40	93.21	97.48	100.00

**Figure 8:**
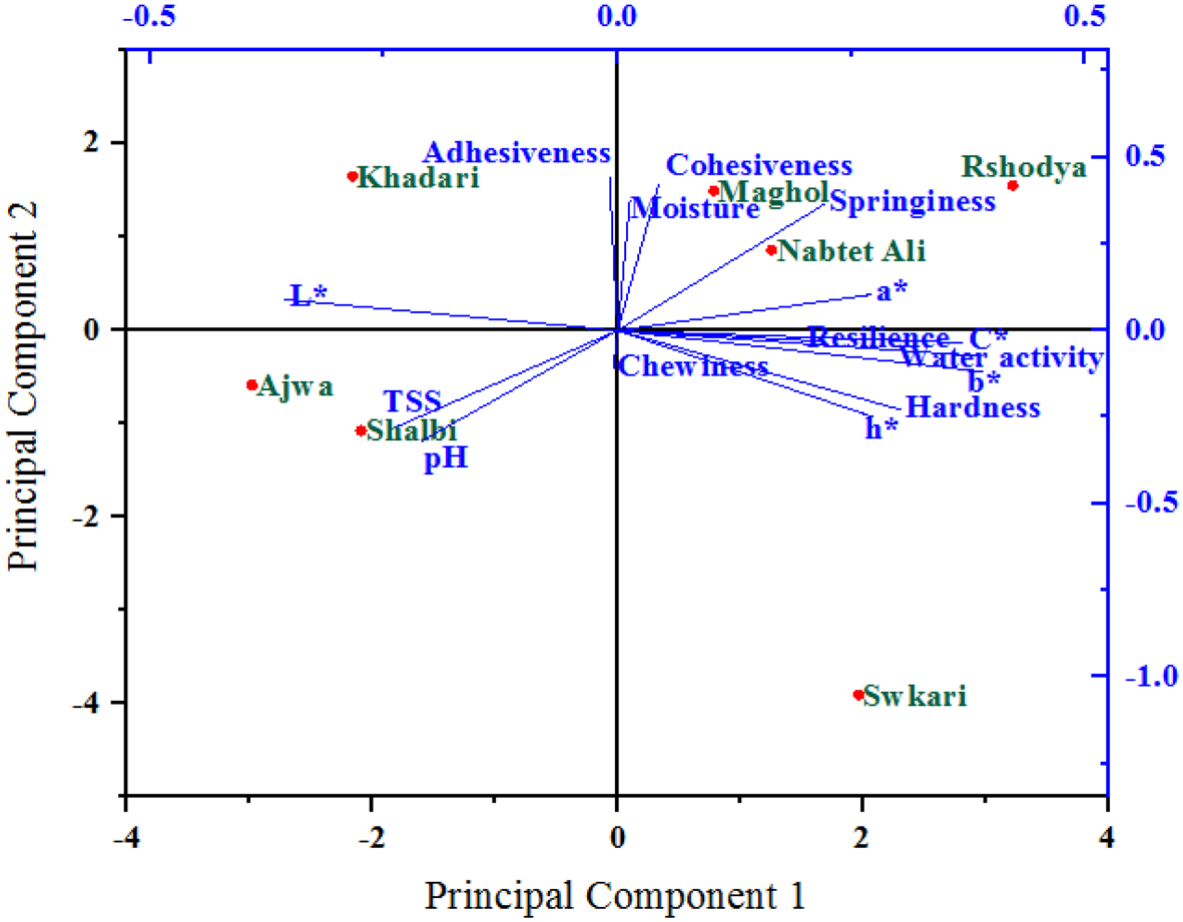
A biplot diagram based on PC1 and PC2 shows similarities and dissimilarities in relationships among the measured variables across different varieties of palm dates under untreated.

**Figure 9:**
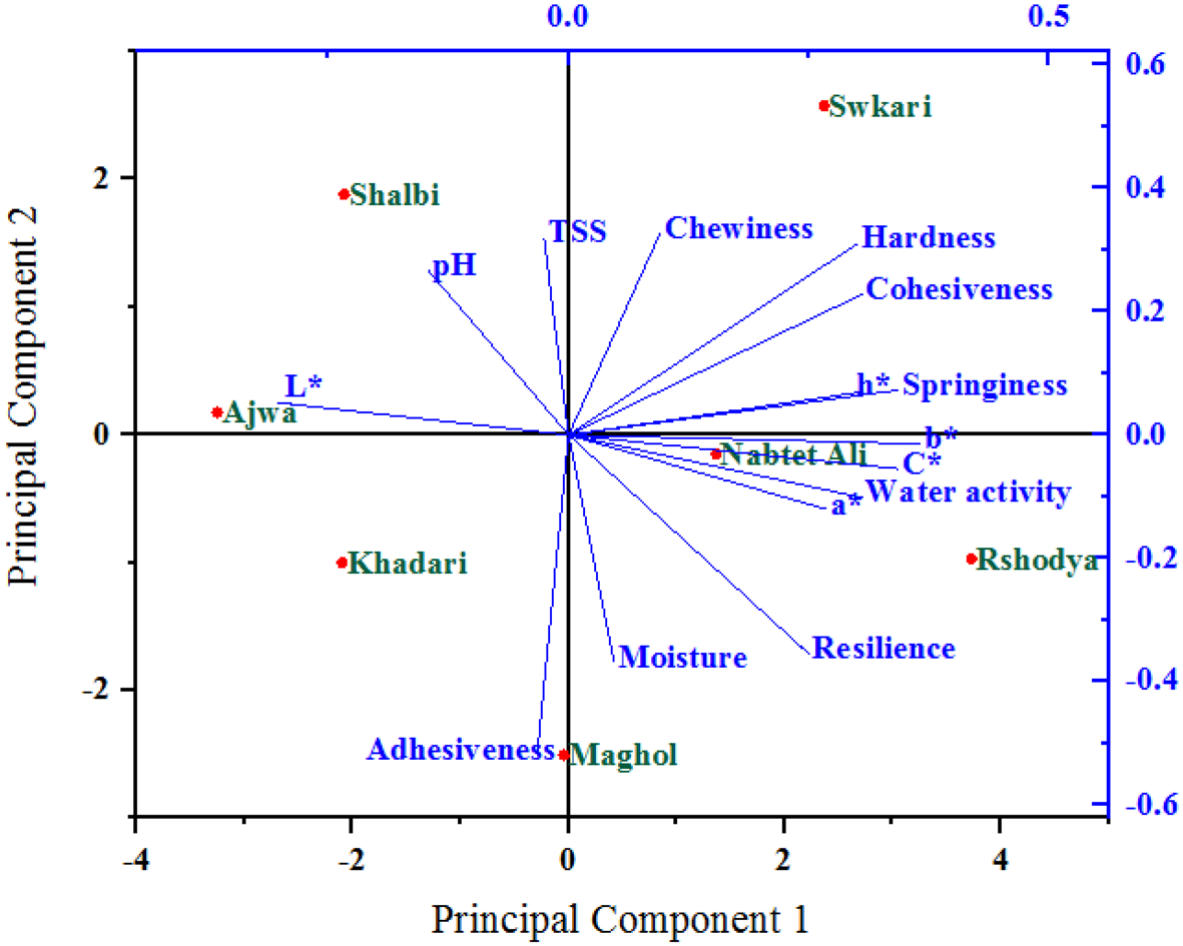
A biplot diagram based on PC1 and PC2 shows similarities and dissimilarities in relationships among the measured variables across different varieties of palm dates under after SDBD treatment.

PCA1 and PCA2 were employed to draw a biplot in order to show the correlation between studied variables under the main effects of untreated (Fig. 8) and treated (Fig. 9) palm date varieties. The PCA1 and PCA2 were positively correlated with most measured variables in both untreated and treated palm date varieties. PCA1 is highly positively correlated with b*, C*, h*, a*, water activity, hardness, springiness, and resilience in both untreated and treated palm date varieties, and with cohesiveness in treated palm date varieties. In untreated palm date types, the PCA2 is strongly positively associated with moisture, cohesiveness, adhesiveness, and springiness, while in treated palm date types, it is strongly correlated with pH, TSS, hardness, chewiness, and cohesiveness. The PCA1 and PCA2 were positively correlated to most quantitative and qualitative traits in the investigated date palm cultivars^77^. A wide range of color and flavor flavonoids and hydroxycinnamates of date palm fruit were found to correlate with PCA1 and some showed no correlation with PCA1^79^. They added, that PCA1 had a strong positive correlation with regulatory polyamines, glutathione-mediated antioxidant activity, energy production, lysophospholipids, amino acids, tannins, non-reducing sugars, and hormones.

A sharp angle was identified among the most measured variables in this investigation in both untreated and treated with SDBD, demonstrating a positive correlation between these variables, but they vary in degree and consistency in quantity. Our findings revealed that most measured variables had sharp angles below 90-degrees, indicating a positive correlation between these variables. The highest and positive correlation (smallest sharp angles) were observed among the most studied variables during each/both treatments. For example, among water activity, hardness, springiness, a*, b*, c* and h* in both treatments. These findings indicate that there are differences between palm date varieties as a result of treatment with plasma. These differences were also higher than in untreated palm dates. Therefore, these variations among these varieties are due to SDBD treatment.

The PCA1 and PCA2 mainly distributed and distinguished the studied variables into four groups according to their degree of correlation in both untreated and treated palm date varieties. Based on the PCA, the results demonstrated substantial variability, and the sixteen quantitative and qualitative qualities evaluated of date palm cultivars revealed a strong discriminating factor^78^. The analyzed variables inside each group were significantly positively or negatively associated with each other, the inverse was true. The first group was related to the highest PCA1 and PCA2 (the first quarter) and included moisture, cohesiveness, springiness, and a* under untreated, as well as cohesiveness, springiness, hardness, chewiness, and h* treated using SDBD, which are strongly positively associated with the varieties *Maghol, Rshodya* and *Nabtet Ali* as well as the *Swkari* variety only, respectively. The second group comprised adhesiveness and L* in untreated as well as pH and L* in treated using SDBD, which were located in the second quarter (the highest PCA2 and the lowest PCA1) with the *Khadari* variety only as well as the two varieties *Shalbi* and Ajwa, respectively. The pH, TSS (untreated), and adhesiveness (treated) in the third quarter formed the third group (the lowest PCA1 and PCA2) with *Ajwa* and *Shalbi* in the control treatment and with *Khadari* and *Maghol* in the SDBD treatment. The other variables in both treatments in the fourth quarter (the fourth group) had the highest PCA1 and the lowest PCA2 and were associated with only *Swkari* variety as well as *Nabtet Ali* and *Rshodya* varieties under untreated and treated using SDBD, respectively. According to the PCA biplot^79^, the *Ajwa* variety was distinguished from the rest of the date palm fruit by moisture, fiber, and protein.

Opposite, there were other relationships between studied variables between groups that were positive (low) or negative, depending on whether the angles between them were acute (large) or obtuse, respectively. It was found that, a correlation between the corresponding metabolite abundance profile of date palm fruit and the PCA scores^80^.

## Conclusions

Non-thermal plasma treatment using SDBD successfully inhibited *A. niger* growth that was extracted from palm date varieties after 3 min treatment. SDBD treatment conditions produced significant changes in physicochemical indexes of palm date varieties, e.g., water activity and, moisture. There were minor variations in the pH values of the palm date varieties after SDBD treatment. The SDBD treatment showed maintenance of color, total soluble solids, and texture characteristics, not inducing any textural change compared with the control. The decrease in moisture content may play a role in inhibiting fungal growth. Therefore, SDBD could be a valuable safe, and quick technique for the decontamination of fungi associated with agricultural products, not only for palm date varieties but it could be utilized for other vegetables and fruits as post-harvest treatment. Regardless of statistical significance, the values of physicochemical properties of palm date varieties changed to a cramped extent. Furthermore, the color indices and the texture parameters of treated palm date varieties were not changed under specific conditions of SDBD treatment. Non-thermal Plasma treatment maintained the quality of the dates, not inducing any chemical composition and/or physical properties change compared with the control treatment, these results had confirmed by the correlation between them, therefore the edibility of SDBD-treated palm dates. The results of PCA analysis could be useful and might help to understand the relation between studied variables of palm date varieties under untreated and treated with SDBD.

## Data Availability

All data generated or analyzed during this study are included in this published article. H_2_O_2_ was obtained as a sdf file from PubChem and converted to a PDB file using PyMOL 2.5 software. The *A. Niger* proteins (PDB: 3K4Q, 1KS5) were obtained from the Protein Data Bank (PDB) (https://www.rcsb.org/structure).
